# Caries preventive efficacy of silver diammine fluoride (SDF) and ART sealants in a school-based daily fluoride toothbrushing program in the Philippines

**DOI:** 10.1186/1472-6831-12-52

**Published:** 2012-11-21

**Authors:** Bella Monse, Roswitha Heinrich-Weltzien, Jan Mulder, Christopher Holmgren, Wim H van Palenstein Helderman

**Affiliations:** 1 Deutsche Gesellschaft für Internationale Zusammenarbeit (GIZ) GmbH, GIZ Office Manila, PDCP Bank Centre, V.A. Rufino cor. L.P. Leviste Str, Makati City, Metro Manila, Philippines; 2 Department of Preventive and Paediatric Dentistry, Jena University Hospital, WHO Collaborating Centre for Prevention of Oral Diseases, Bachstr. 18, 07743, Jena, Germany; 3 Department of Global Oral Health, College of Dental Sciences, Radboud University Nijmegen Medical Centre, 6500 HB, Nijmegen, The Netherlands; 4 Aide Odontologique Internationale, 1 Rue Maurice Arnoux, 92120, Montrouge, France; 5 Dental Health International Nederland (DHIN), Korte Linschoten OZ 14, 3461 CG, Linschoten, The Netherlands

**Keywords:** Silver diamine fluoride, Toothbrushing, Sealants, Permanent molars, Dental caries

## Abstract

**Background:**

Occlusal surfaces of erupting and newly erupted permanent molars are particularly susceptible to caries.

The objective of the study was to assess and compare the effect of a single application of 38% SDF with ART sealants and no treatment in preventing dentinal (D3) caries lesions on occlusal surfaces of permanent first molars of school children who participated in a daily school-based toothbrushing program with fluoride toothpaste.

**Methods:**

The prospective community clinical trial in the Philippines was conducted over a period of 18 months and included 704 six- to eight-year-old school children in eight public elementary schools with a daily school-based fluoride toothpaste brushing program. Children were randomly assigned for SDF application or ART sealant treatment. Children from two of the eight schools did not receive SDF or ART sealant treatment and served as controls. SDF or ART sealant treatment was applied on sound occlusal surfaces of permanent first molars. Surfaces that were originally defined as sound at baseline but which changed to dentinal (D3) caries lesions were defined as surfaces with new caries (caries increment). Non-compliance to the daily toothbrushing program in three schools offered the opportunity to analyze the caries preventive effect of SDF and sealants separately in fluoride toothpaste brushing and in non-toothbrushing children.

**Results:**

In the brushing group, caries increment in the SDF treatment group was comparable with the non-treatment group but caries increment in the sealant group was lower than in the non-treatment group with a statistically significant lower hazard ratio of 0.12 (0.02-0.61). In the non-brushing group, caries increment in the SDF treatment group and the sealant group was lower than the non-treatment group but the hazard ratio was only statistically significant for the sealant group (HR 0.33; 0.20-0.54). Caries increment was lower in toothbrushing children than in non-toothbrushing children. Hazard ratios reached statistical significance for the non-treated children (HR 0.43; 0.21-0.87) and the sealant-treated children (HR 0.15; 0.03-0.072).

**Conclusions:**

A one-time application of 38% SDF on the occlusal surfaces of permanent first molars of six- to eight-year-old children is not an effective method to prevent dentinal (D3) caries lesions. ART sealants significantly reduced the onset of caries over a period of 18 months.

**Trial registration number:**

German Clinical Trial Register DRKS00003427

## Background

Dental caries is a global pandemic [1,2]. Treatment of caries in children is virtually non-existent in a number of low- and middle-income countries and, in children under six years of age, it is limited even in many high-income countries [3]. The situation in the Philippines, a low-middle-income country, is no different. According to the 2006 Philippine National Oral Health Survey, 97% of the six-year-old children and 82% of the 12-year-olds suffer from dental caries and the few restorations observed indicate that restorative treatment is rather rare [4]. In both age groups just over 40% of existing caries lesions have progressed to odontogenic infections [5]. Furthermore, 20% of 6-year-olds and 16% of 12-year-olds reported having a problem in their mouth at the time of being questioned [4]. According to the Department of Education, the principal reason for absenteeism from school in the Philippines is toothache [6]. Dental caries in children appears to be the most prevalent childhood disease in the Philippines followed by soil-transmitted helminthiasis [7]. Moreover, the presence of odontogenic infections in 12-year-olds appears to be associated with a low Body Mass Index (BMI) and this association might represent a significant yet largely neglected determinant of poor child development [8].

The dramatic decline in caries over the past three decades, seen in many high-income countries, is largely attributed to the widespread use of fluoride toothpaste, in spite of continued consumption of high levels of sugar [9,10]. There are no mass fluoridation schemes in the Philippines and the high caries experience in Filipino children suggests that the use of fluoride toothpastes with anti-caries efficacy is minimal, although no reliable data exist. This emphasizes the urgent need for appropriate exposure to fluoride in the country, which should be an essential component of any preventive oral health care program [11-14]. The Global Consultation on Oral Health through Fluoride [12], which was jointly convened by the World Health Organization (WHO), the FDI World Dental Federation (FDI) and the International Association for Dental Research (IADR) in November 2006 in Geneva stated that “Prevention by using fluoride is the only realistic way of reducing this burden (of dental caries) in populations”. In addition, the WHO, in 2007, at the 60th World Health Assembly [13] urged governments “to promote oral health in schools, aiming at developing healthy lifestyles and self-care practices in children” while the declaration of the Beijing conference in 2007 [14] which was jointly convened by the WHO, the FDI, the IADR and the Chinese Stomatological Association (CSA) stated that “fluoride toothpaste remains the most widespread and significant form of fluoride used globally and the most rigorously evaluated vehicle for fluoride use”.

In 2004, several years before these international recommendations were proclaimed, a pilot project was initiated in the Philippines consisting of daily school-based toothbrushing with fluoride toothpaste for the children of eight elementary public schools in Cagayan de Oro (Mindanao) and Manila.

It was anticipated that daily school-based toothbrushing with fluoride toothpaste, starting in grade 1 schoolchildren, would impact on the caries levels of the permanent dentition [15]. It was also hoped that a single application of 38% silver diammine fluoride (SDF) would provide additional caries preventive effect over and above that provided by the regular use of fluoride toothpaste. Any additional caries preventive effect would be particularly useful to tide over periods of high caries susceptibility e.g. during the period of eruption of first permanent molars [16]. Even though the literature indicates that any fluoride application over a period of 2–3 years, be it a varnish, a fluoride gel or fluoride rinse results only in a modest additional caries preventive effect of about 10% when used together with daily toothbrushing with fluoride toothpaste [17], SDF applications were not included in this systematic review. One study has shown promising results with SDF on caries prevention [18], while a systematic review concluded that SDF was more effective than fluoride varnish in preventing caries [19]. The rationale for the selection of 38% SDF as an additional preventive measure was its ease and speed of application, its low cost, and its potentially high caries-preventive and caries-arresting effect [18,19]. Moreover, this intervention was also selected in the perspective of being a feasible component in a national school based health program. A single application of SDF was chosen because repeated applications were unlikely to be feasible in the resource-limited setting of the Philippines.

Another, potentially more costly and more time consuming approach, is the provision of sealants. Studies have shown the effectiveness of sealants to prevent occlusal caries, and in the Philippine school setting, where dental facilities are not available, ART glass-ionomer cement sealants applied with the finger-press technique were considered the most appropriate sealant method [20]. These sealants placed on occlusal surfaces of permanent first molars of first graders were considered a positive control for the single 38% SDF application.

The aim of the present study therefore was to assess and compare the effect of a single application of 38% SDF with ART sealants and no treatment in preventing dentinal (D3) caries lesions on occlusal surfaces of permanent first molars of first graders of the eight public elementary schools that participated in a daily school-based toothbrushing program with fluoride toothpaste.

## Methods

This study, which started in 2004, was a community clinical trial on a cohort of first graders of eight public elementary schools to evaluate the effect of treatment under prevailing contextual factors of low-income countries.

### Study population

**Figure 1:**
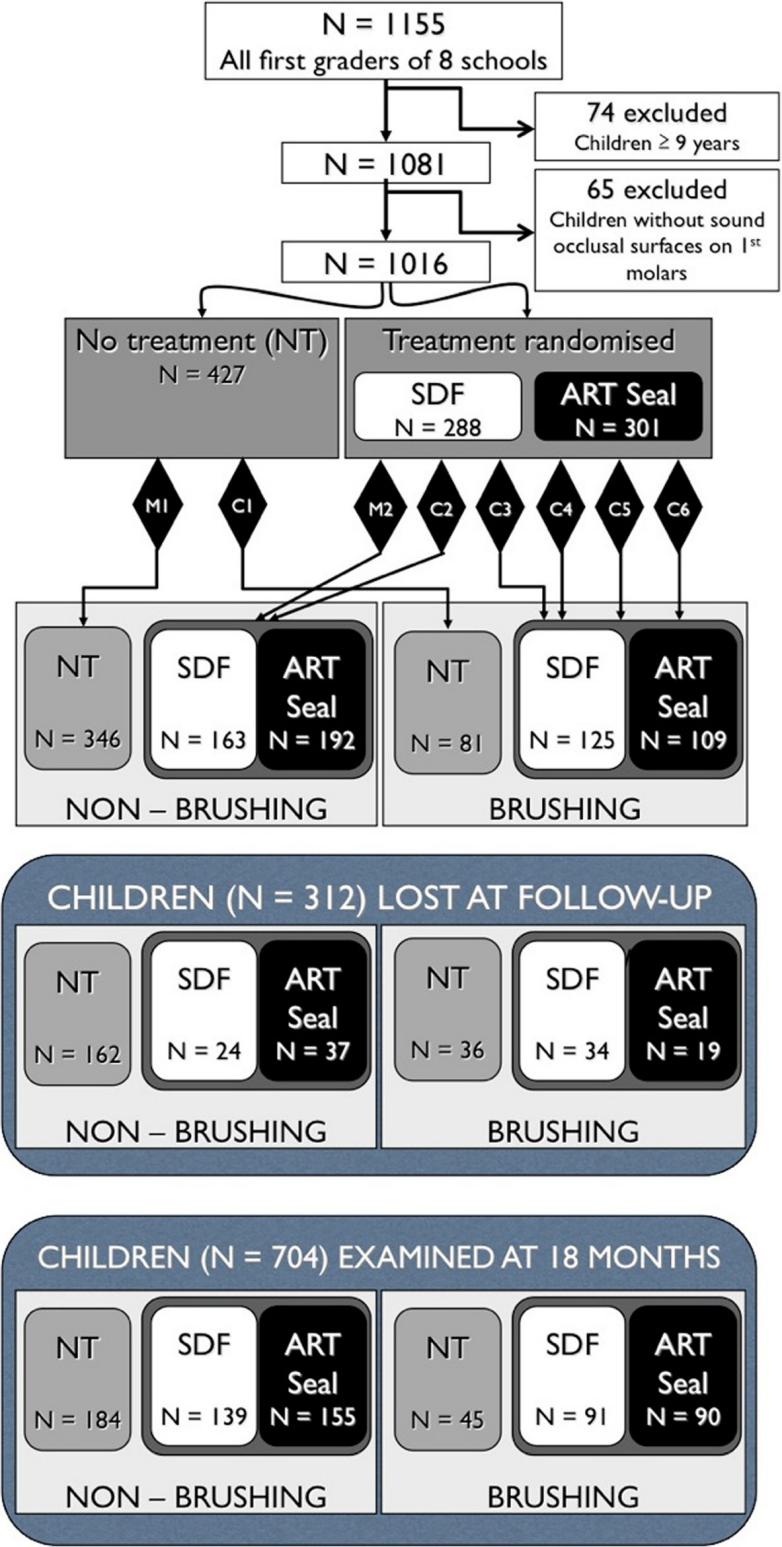
Flow chart of the study including participating schools, groups and students (M1 and M2 represent schools in Manila, C1 to C6 represent schools in Cagayan de Oro).

Six public elementary schools were selected in Cagayan de Oro and two in Manila, in the Philippines. All eight schools were involved in an on-going oral health care program that included daily school-based toothbrushing with fluoride toothpaste (1450 ppm F) that had been implemented in the months immediately preceding the start of this study. All first graders (n=1155) were examined. Inclusion criteria for the study were children of six to eight years of age in grade one who had at least one erupted permanent first molar with a sound occlusal surface. “Sound“included clinically sound and all stages of enamel caries up to but not including visible dentinal (D3) caries lesions. Of the excluded children, 74 (6.4%) were nine years or older and 65 (5.6%) children did not meet the inclusion criterion of having at least one erupted permanent molar with a sound occlusal surface (Figure 1). All excluded first graders (n=139) received SDF treatment on decayed surfaces of the permanent dentition. Tooth extraction was offered to all first graders in cases where the caries lesion had progressed into the pulp. One of the two schools in Manila and one of the 6 schools in Cagayan de Oro were appointed by lottery as control schools (no treatment). In the remaining six schools all first graders meeting the inclusion criteria received treatment. Children with a consecutive odd number on the class register received an SDF application and those with a consecutive even number ART sealants on sound occlusal surfaces of their permanent first molars. Children assigned for SDF treatment also received an SDF application on decayed surfaces of the permanent dentition while children assigned for ART sealants received ART restorations on decayed (D3) surfaces of the permanent dentition.

Signed informed consent was obtained from the parents of all the first graders of the selected schools. The ethics commission of Xavier University, Cagayan de Oro, Philippines, approved the study protocol.

Based on an assumed 10% reduction in caries increment in the treated group as compared to the non-treatment group, a statistical power of 80%, a p-value of 0.05 and an anticipated drop out of 35%, with 1016 included children a necessary sample size of at least 200 children per group was possible (Table 1).

**Table 1:** Baseline caries experience on occlusal surfaces of permanent first molars per study group (n=1016)

**All 8 schools**						
	**Children N**	**Age (sd)**	**Surfaces N %**			
			**Total**	**Missing**	**Sound**	**D3 caries**
Non-treated	427	6.7 (0.7)	1708	181 10.6%	1257 73.6%	270 15.8%
SDF treated	288	6.7 (0.7)	1152	142 12.3%	881 76.5%	129 11.2%
Sealants	301	6.7 (0.7)	1204	110 9.1%	951 79.0%	143 11.9%
Total	1016	6.7 (0.7)	4064	433 10.7%	3089 76.0%	542 13.3%

### Examination

The examinations were performed outside in the schoolyard with sunlight as a direct light source. The children were placed in a supine position on a long classroom bench, with their heads on a pillow on the lap of the examiner, who sat behind them. Teeth were examined after drying with cotton rolls and the occlusal surface of the permanent first molars were additionally dried with cotton pellets. A CPI ball-end probe and mouth mirror were used as examination tools and caries was scored according to procedures described by WHO [21]. Sound was scored except when: 1) the CPI probe could enter a caries cavity indicating a dentinal (D3) caries lesion or 2) if the probe could not enter a small discontinuity in the enamel but a greyish appearance of the enamel was seen as a sign of undermined caries indicating a dentinal (D3) caries lesion.

### Calibration of examiners

Children were examined at baseline and followed up by eight calibrated examiners. A WHO consultant undertook training and calibration of examiners over a three-day period at one local school that was not involved in the study. Intra-examiner reproducibility was assessed on 10% of the children during the baseline and the follow-up examination.

### Treatment regimens

SDF treatment was provided by school nurses who had received a one-day training in the technique and who worked under the supervision of a dentist. Sealants were placed by dentists of the Department of Education who had received training in the provision of ART glass-ionomer cement sealants by the supplier of the material. All treatment was provided with chairside assistance. Prior to treatment a school nurse brushed all permanent first molars without toothpaste.

School children in the SDF group received one application of 38% SDF solution (Saforide, Bee Brand Medical, Japan) on sound occlusal surfaces of all erupted permanent first molars (“sound” as defined previously). Molars were isolated with cotton rolls. Cotton pellets were then used to dry the occlusal surface. SDF was applied on the occlusal surface by rubbing for 1 minute with a Vivabrush (Ivoclar Vivadent GmbH, Liechtenstein). Next, tannic acid (strong black tea) was applied to precipitate the SDF [22]. Excess was removed with a dry cotton pellet. Thereafter, a layer of Vaseline was applied to protect the SDF from saliva. Children were asked not to eat for one hour after treatment.

Children in the sealant group received ART glass-ionomer cement sealants on sound occlusal surfaces of all erupted permanent first molars. The treatment was provided according to the ART sealant application procedure [23]. A high-viscosity material (Ketac Molar Easymix, 3M ESPE, Germany) was used strictly according to the manufacturer’s instructions.

### Change in the study design due to non-compliance to the daily school-based toothbrushing program with fluoride toothpaste

During regular monitoring visits to the schools, following the implementation of the daily school-based toothbrushing with fluoride toothpaste program, within a month it became apparent that one school in Cagayan de Oro and two schools in Manila were not complying with the program. Since the study was already in progress, it was decided to maintain these three schools in the research program for evaluation at 18 months. The analysis of 18-month data on the effect of SDF and ART sealants was therefore divided into school children with daily school brushing with fluoride toothpaste and those without. The children in the non-compliant schools continued to receive a yearly distribution of one toothbrush and one sachet of fluoride toothpaste and a lecture on oral health as is routinely provided to all school children in the Philippines.

### Blinding of the examiners

The eight examiners undertaking the evaluation were not involved in the treatment. They were informed about the presence of teeth with an ART sealant or ART restoration since they are often clinically indistinguishable. They were however blinded as to whether subjects had received SDF treatment or not, or whether the school was compliant with the daily toothbrushing program with fluoride toothpaste.

The evaluation was carried out after 18 months using the same examination setting, criteria, tools and examiners.

### Statistical analysis

The data were analyzed with SAS 9.1 software. Intra-examiner reproducibility at tooth level at baseline and at follow-up examinations was calculated with Kappa statistics. The baseline data regarding percentages between the different groups were analyzed with chi-square tests. For the analysis on the development of new dentinal (D3) caries lesions (caries increment) on sound occlusal surfaces of first permanent molars, a Cox proportional hazard model was applied [24] taking into account frailties correction for the child as a unit of analysis [25].

## Results

Unweighted intra-examiner mean Kappa at baseline examination for caries was 0.90 and varied between 0.86 and 0.97 while the intra-examiner mean Kappa at follow-up examination was 0.93 (0.91-0.96).

The flow chart (Figure 1) shows the number of children at baseline (n=1016) that met the inclusion criteria. None of the children had restorations in their permanent first molars. The chart also presents the number of children in the SDF, sealant and non-treatment group and the subsequent sub-division of these children into brushing and non-brushing groups. Finally the number of dropouts is presented of the six different groups and the number of children available (n=704) for the 18-months evaluation. Table 1 presents the number of children at baseline (n=1016), the age, the number and percentage of missing molars (unerupted) and sound occlusal surfaces and decayed (D3) occlusal surfaces according to study group. The only statistical significance between the three groups was seen in the non-treatment group that had a higher percentage of D3 occlusal surfaces (Chi square, p value <0.02). The baseline data for the dropouts (Table 2) did not differ in statistically significant ways from those who entered the study at baseline (Table 1). Table 3 presents the baseline data of children remaining in the study (n=704). The caries level at baseline of children in the non-treatment group was higher than those in the SDF or ART sealant group, but the difference only reached statistical significance in the toothbrushing group (Chi square, p<0.02).

**Table 2:** Baseline caries experience on occlusal surfaces of permanent first molars of children lost to follow up per study group (n=312)

**Brushing**						
	**Children N**	**Age (sd)**	**Surfaces N %**			
			**Total**	**Missing**	**Sound**	**D3 caries**
Non-treated	36	6.6 (0.7)	144	23 16.0%	102 70.8%	19 13.2%
SDF treated	34	6.7 (0.8)	136	14 10.3%	103 75.7%	19 14.0%
Sealants	19	6.8 (0.9)	76	4 5.3%	60 79.0%	12 15.8%
Total	89	6.7 (0.8)	356	41 11.5%	265 74.4%	50 14.0%
**Non-brushing**						
Non-treated	162	6.8 (0.7)	648	52 8.0%	484 74.7%	112 17.3%
SDF treated	24	6.8 (0.7)	96	10 10.4%	72 75.0%	14 14.6%
Sealants	37	6.8 (0.7)	148	10 6.8%	123 83.1%	15 10.1%
Total	223	6.8 (0.7)	892	72 8.1%	679 76.1%	141 15.8%

**Table 3:** Baseline caries experience on occlusal surfaces of permanent first molars of children per group who participated in the 18 months evaluation (n=704)

**Brushing**						
	**Children N**	**Age (sd)**	**Surfaces N %**			
			**Total**	**Missing**	**Sound**	**D3 caries**
Non-treated	45	6.8 (0.6)	180	24 13.3%	127 70.6%	29 16.1%
SDF treated	91	6.7 (0.7)	364	43 11.8%	288 79.1%	33 9.1%
Sealants	90	6.7 (0.6)	360	23 6.4%	301 83.6%	36 10.0%
Total	226	6.7 (07)	904	90 10.0%	716 79.2%	98 10.8%
**Non-brushing**						
Non-treated	184	6.6 (0.6)	736	82 11.1%	544 73.9%	110 14.9%
SDF treated	139	6.7 (0.6)	556	75 13.5%	418 75.2%	63 11.3%
Sealants	155	6.7 (0.6)	620	73 11.8%	467 75.3%	80 12.9%
Total	478	6.7 (0.6)	1912	230 12.0%	1429 74.7%	253 13.3%

Surfaces that were originally defined as sound at baseline but which changed to dentinal (D3) caries lesions actually indicated the caries increment since no restorations were found on these surfaces. Table 4 depicts the caries increment of the six different groups at the 18-months evaluation.

**Table 4:** Number of sound surfaces, number of surfaces with new dentinal caries (D3) lesions and caries increment after 18 months according to group

	**Brushing**			**Non-brushing**		
	**Non-treated N =127**	**SDF treated n = 288**	**Sealants n = 301**	**Non-treated n = 544**	**SDF treated n = 418**	**Sealants n = 467**
Sound (n)	117	262	298	453	366	438
New dentinal (D3) caries (n)	10	26	3	91	52	29
Caries increment	0.08	0.09	0.01	0.17	0.12	0.06

In the toothbrushing group, caries increment for those who received SDF treatment was comparable to the non-treatment group but caries increment in the sealant group was substantially lower than in the non-treatment group with a statistically significant lower hazard ratio of 0.12 (0.02-0.61) (Table 5). In the non-brushing group, caries increment in the SDF treatment group and the sealant group was lower than the non-treatment group but the hazard ratio was only statistically significant for the sealant group (HR 0.33; 0.20-0.54). Caries increment was lower in toothbrushing children than in non-toothbrushing children. Hazard ratios reached statistical significance for the non-treated children (HR 0.43; 0.21-0.87) and the sealant-treated children (HR 0.15; 0.03-0.072).

**Table 5:** Hazard ratios (95% CI) and p-values between treatment and non-treatment in brushing and non-brushing schools and between brushing and non-brushing schools

**Groups comparison (n=surfaces)**		**Hazard ratio (95% CI) and p-value**
**Brushing schools**		
SDF treated (n=288)	versus non-treated (n=127)	1.16 (0.51-2.63) p=0.72
Sealants (n=301)	versus non-treated (n=127)	0.12 (0.02-0.61) p<0.01
**Non-brushing schools**		
SDF treated (n=418)	versus non-treated (n=544)	0.71 (0.45-1.11) p=0.12
Sealants (n=467)	versus non-treated (n=544)	0.33 (0.20-0.54) p<0.001
**Brushing**	**versus non-brushing**	
Non-treated brushing (n=127)	versus non-treated non-brushing (n=544)	0.43 (0.21-0.87) p<0.02
SDF treated brushing (n=288)	versus SDF treated non-brushing (n=418)	0.70 (0.38-1.27) p=0.23
Sealants brushing (n=298)	versus sealants non-brushing (n=438)	0.15 (0.03-0.72) p<0.02

The retention rate after 18 months was 58% for complete and partially retained sealants and 42% for those that were totally missing. Retention rates differed between the toothbrushing and non-toothbrushing group but not statistically significantly.

## Discussion

Practical considerations and unforeseen events that took place during the study meant that this study had several methodological limitations that unfortunately lower the level of evidence of the findings. Studies in developing countries have to deal with the prevailing realities where methodological considerations often have to succumb to political and resource demands. For instance, the selection of participating schools, six in Cagayan de Oro and two in Manila were from a methodological point of view less than optimal. However, this resulted from an insistence of the Central Office of the Department of Education (DepEd) that the study be undertaken not only in the city of Cagayan de Oro but also in the capital city of Manila. The distances incurred between the study locations therefore obliged a greater number of examiners than would be ideal. For this reason it was not practical to undertake inter-examiner’s reproducibility in any meaningful way. Another methodological problem was the assignment of non-treatment (control) children. The intention was to randomize the first graders of the eight schools into those who received treatment and those who received no treatment. Since the treatment was provided on the school premises, the principals and local educational officers did not authorize a design where certain children in the same school would serve as non-treatment controls. Separate schools therefore had to be used for the non-treatment controls.

Randomized controlled study designs have become the ‘gold standard’ in research [26]. However, it has been argued that the application of such high quality study design is often logistically impossible and unaffordable owing to limited recourses [27]. Researchers are often confronted with such difficulties when conducting community-based research in countries with limited resources. Nevertheless, we believe that conducting clinical research in countries where oral health problems are high is essential since it is not always appropriate or relevant to transfer study findings from developed countries to developing countries. It has been questioned if randomized controlled trials conducted under optimal conditions are able to address questions of effectiveness and efficiency of clinical interventions in health care systems adequately [27], because of the problems of applicability and transferability from controlled trial conditions to real-life conditions in communities.

The current study of 18 months duration, although with limitations, has already shown important findings. It would, however, be unwise to speculate on the longer-term results from the different groups.

The affluent upper and middle class in the Philippines educate their children in private schools. The remaining Filipino child population (about 90%) attend public elementary schools because their parents cannot afford the school fees of private schools. Differences in oral health of these children between rural and urban areas are not apparent and all tend to have comparable socio-economic backgrounds [4]. Nevertheless, differences in caries levels were seen at baseline between groups. The existing bias at baseline was, however, to a certain extent bypassed by analyzing caries increment over the 18-months period.

The high mobility of people in the Philippines and frequent school absenteeism due to illness result in large dropout rates of children in elementary schools. The loss to follow-up rate in this study was in line with the official dropout rates in rural Mindanao, which are known to be the highest between grade one and grade two. This has to be taken into account when clinical research is planned.

The unforeseen lack of compliance with daily school toothbrushing in three of the study schools is an example of the difficulties encountered in such studies, but it allowed for the opportunity to conduct a further evaluation on the background effect of lack of brushing with fluoride toothpaste on the applied treatment regimes. The consequence of dividing the whole sample impacts negatively on the power of this study and unfortunately limits the interpretation of data.

A two-year school-based daily fluoride toothpaste brushing program in five-year-old school children in Scotland with high caries risk showed a 56% reduction in caries increment of the permanent first molars [28]. In Indonesia, a three-year school-based toothbrushing program with fluoride toothpaste in elementary schools resulted in up to a 40% reduction in caries [29]. The hazard ratio of 0.43 (CI; 0.21-0.87) for new caries development in the brushing groups with fluoride toothpaste is therefore consistent with results achieved from other school-based toothbrushing programs with fluoride toothpaste in deprived communities.

The sealants that were applied in the present study could be considered as both preventive sealants (on surfaces clinically free of caries) and therapeutic sealants on surfaces with all stages of enamel caries lesions up to but not including visible dentinal caries. Unfortunately, despite attempts to do so, it was impossible to distinguish between sound and the different stages of enamel caries lesions that precede dentinal caries in the present study since these stages of caries could not reliably be diagnosed in the described field setting in the absence of compressed air, suction and proper lighting.

The retention rates of ART sealants in this study were moderate. Other studies have reported retention rates of high viscosity GIC sealants that vary considerably [30-33]. This variance may be explained by the clinical conditions (field setting) under which sealants are placed, the teeth selected for sealing, operator factors and inadequate training.

This study assessed the effect of SDF on preventing dentinal caries lesions but it was not designed to determine the degree of arrested caries that has been reported in other controlled clinical trials [18,22]. Only one controlled clinical trial in Cuba on 38% SDF application for the prevention of new caries (D3) on occlusal surfaces of permanent first molars has been published [18]. After three years of biannual application of SDF, a significant caries reduction of 64% was reported, but the results are difficult to interpret, since most of the occlusal surfaces in the control group had received restorations. The present study is the first clinical trial that assesses the caries preventive efficacy of SDF application in children who brushed daily at school with fluoride toothpaste and is the second study after that of Yee et al. [22] that evaluates the effect of a single application of 38% SDF. In common with this last study, tannic acid was used as an inexpensive reducing agent after SDF application to accelerate the deposition of silver phosphate [34]. Recently, it has been shown that this extra step might not be necessary, since tannic acid did not appear to have any additional effect [22]. The present study showed no additional caries preventive effect, 18 months after a single application of 38% SDF on permanent first molars of six- to eight-year-old children who brushed their teeth daily at school with fluoride toothpaste. It is also questionable whether SDF has any efficacy in preventing new caries in children who are not under a toothbrushing with fluoride toothpaste regime. The present finding urges more research on the efficacy of SDF on the onset of new caries.

## Conclusions

In this field study, conducted in Filipino elementary schools, a one-time application of 38% SDF on the occlusal surfaces of permanent first molars of six- to eight-year-old children was not an effective method to prevent the onset of new dentinal (D3) caries lesions in children. ART sealants significantly reduced the onset of new dentinal (D3) caries lesions over a period of 18 months.

## Competing interests

The authors declare that they have no competing interests.

## Authors’ contributions

BM was the principal investigator and prepared the first draft of the paper. JM performed the statistical data analysis, WvP contributed to the critical interpretation of the data. RHW and CH were involved in the data collection and interpretation. All authors contributed equally to the final version of the paper and have read as well as approved the final manuscript.

## Authors' information

BM worked in the frame of the ‘integrated expert program’ of the German Development Cooperation as advisor to the Department of Education and supported the Department in the development of low-cost strategies to improve oral health of children in the Philippines.

## Pre-publication history

The pre-publication history for this paper can be accessed here:

http://www.biomedcentral.com/1472-6831/12/52/prepub
